# Supplementary material for: Early Risk Score for Predicting Hypotension in Normotensive Patients with Non-Variceal Upper Gastrointestinal Bleeding
Source: J Clin Med. 2019 Jan 2;8(1):37. doi: 10.3390/jcm8010037 (PMC6352164; doi:10.3390/jcm8010037)
Supplement: Supplementary file 1 [file jcm-08-00037-s001.pdf]

Table S1. Outcomes of the patients in the development group

Outcomes	Total (N= 1046)
Endoscopic hemostasis, n (%)	342 (32.6)
RBC transfusion, n (%)	593 (56.6)
Amount of RBC transfusion, median (IQR)	2.0 (0.0 – 3.0)
Embolization, n (%)	34 (3.3)
Surgery of bleeding control, n (%)	22 (2.1)
Hypotension occurrence within 24 h, n (%)	98 (9.4)
In-hospital mortality, n (%)	10 (1.0)
Thirty day mortality, n (%)	14 (1.4)
Rebleedig, n (%)	100 (9.6)
ICU admission, n (%)	31 (3.0)
Hospital stay, median (IQR)	3.0 (2.0 – 6.0)
Risk scores, median (IQR)	
GBS	10.0 (7.0 – 13.0)
Pre-endoscopy Rockall	1.0 (1.0 – 3.0)

GBS Glasgow-Blatchford score, *ICU* intensive care unit, *IQR* interquartile range, *RBC* red blood cell

Table S2. Endoscopic finding of the patients in the development group

Endoscopic finding, n (%)	Data
Gastric ulcer	271 (25.9)
Erosive gastritis	161 (15.4)
Duodenal ulcer	124 (11.9)
Mallory-Weiss syndrome	63 (6.0)
Malignant cancer	61 (5.8)
Erosive esophagitis	46 (4.4)
Dieulafoy’s lesion	32 (3.1)
Variceal bleeding	31 (2.9)
Arteriovenous malformation	19 (1.8)
Erosive duodenitis	4 (0.4)
Others	69 (6.6)

Table S3. Test of accuracy of cutoff value for predicting need of red blood cell transfusion in the validation set.

Score	Number of hypotension	Sensitivity (%)	Specificity (%)	PPV (%)	NPV (%)	AUC
New score (≤2)	76	62.7	66.7	67.0	62.4	0.647
GBS (≥7)	181	88.7	62.4	71.8	83.7	0.756

GBS Glasgow-Blatchford Score, *NPV* negative predictive value, *PPV* positive predictive value
